# Supplementary material for: Prognostic Factors for Staying at Work for Partially Sick-Listed Workers with Subjective Health Complaints: A Prospective Cohort Study
Source: Int J Environ Res Public Health. 2020 Sep 30;17(19):7184. doi: 10.3390/ijerph17197184 (PMC7578951; doi:10.3390/ijerph17197184)
Supplement: Supplementary file 1 [file ijerph-17-07184-s001.pdf]

Table 1. General questions and collected data from the Dutch Social Security Institute.

Domains	Categories/ Ranges	General questions from the questionnaires at baseline (T0)	Data from the Dutch Social Security Institute
**Participants' demographics**			
Age in years	18-65	X	X
Gender	Male / Female	X	X
Marital status (married or partner)	Yes / No	X	X
Breadwinner of the family	Yes / No	X	
Land of birth	The Netherlands / Other country	X	
Educational level	Primary or Secondary school / High school / Bachelor's or Master's degree	X	X
**Participants' socio-economic and work-related status**			
Collar job	Blue / White / Pink	X	
Employed	Yes / No	X	
Usual working time in hours	4-60	X	
Regular work schedule	Yes / No	X	
Managerial position	Yes / No	X	
Job demands	Psychological / Physical / Combination of both	X	
Previous absenteeism for the same health complaint	Yes / No	X	
Work disability benefits	No or Partial / Full		X
Adjustments at work	Yes / No	X	
Interventions at work (e.g., job coaching)	Yes / No	X	
**Participants' health**			
Use of specialist care in the last 2 years	Yes / No	X	
Use of psychiatric care in the last 2 years	Yes / No	X	
Use of medication	Yes / No	X	
Subjective health complaints (SHC)	Yes / No		X
**Participants' self-perceived ability**			
Positive expectations for staying at work	Yes or Inconclusive / No	X	

Figure 1. Multicollinearity analyses for workers with subjective health complaints (SHC).

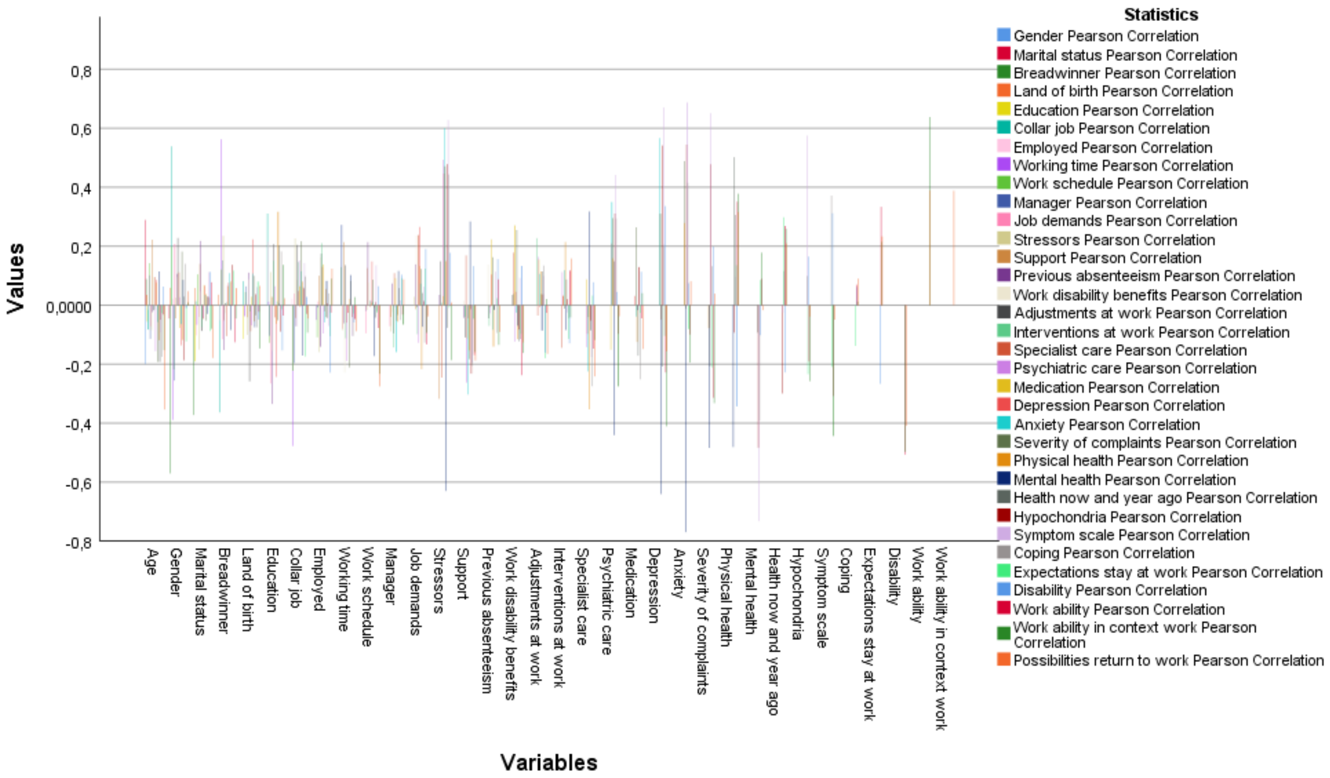

Table 2. Variance inflation factor (VIF) multicollinearity scores for workers with subjective health complaints (SHC).

Variables	VIF score
Age	1.70
Gender	3.66
Marital status	2.10
Breadwinner	4.41
Land of birth	1.76
Education	2.15
Collar job	2.65
Employed	1.59
Working time	3.91
Work schedule	1.58
Managerial position	1.70
Job demands	1.84
Stressors	4.32
Support	1.76
Previous absenteeism for the same health complaint	1.58
Work disability benefits	1.73
Adjustments at work	1.99
Interventions at work	1.66
Specialist care	1.64
Psychiatric care	2.16
Medication	1.90
Depressive disorder	4.98
Anxiety disorder	4.53
Severity of complaints	3.62
Physical health	4.40
Mental health	9.38
Health compared to a year ago	2.24
Hypochondria	2.71
Symptom scale	6.47
Coping	2.22
Expectations staying at work	1.95
Disability	2.43
Work ability in general	3.39
Work ability in the context of work load	3.25
Possibilities for returning to work	2.43

Table S3. Missing data analyses of the baseline characteristics of the present study population

		Cases with missing outcome[1] (No[2]=87)		Cases with no missing outcome[3] (No=571)		Chi-square or T-test
**Domains**	**Categories/ Ranges**	**Mean /No**	**SD[4]/ %**	**Mean /No**	**SD/%**	***p***
**Demographic**						
Age in years	18-65	48.46	10.06	50.49	9.50	0.07
Gender	Male	42	48%	261	46%	0.66
Marital status	Married or partner	54	63%	426	75%	0.02
Breadwinner of the family	Yes	65	75%	379	67%	0.13
Land of birth	The Netherlands	75	86%	520	91%	0.15
Educational level	Primary / Secondary school	42	48%	225	39%	0.21
	High school	29	34%	191	34%	-
	Bachelor's / Master's degree	15	18%	152	27%	-
**Socio-economic and work-related**						
Collar job	Blue	27	32%	119	21%	0.04
	White	30	37%	204	37%	-
	Pink	25	31%	236	42%	-
Employed	Yes	73	85%	500	89%	0.27
Usual working time in hours	4-60	32.43	8.77	32.71	8.72	0.78
Regular work schedule	Yes	56	64%	405	71%	0.21
Managerial position	Yes	13	15%	82	14%	0.89
Job demands	Psychological	20	23%	189	33%	0.17
	Physical	33	38%	187	33%	-
	Combination of both	34	39%	195	34%	-
Stressors[5]	16-64	35.53	8.77	35.67	8.56	0.89
Support[5]	21-84	59.60	13.00	61.26	11.53	0.22
Previous absenteeism for the same health complaint	Yes	35	41%	288	51%	0.08
Work disability benefits	No / Partial	67	77%	424	74%	0.58
Adjustments at work	Yes	66	76%	464	81%	0.49
Interventions at work (e.g., job coaching)	Yes	71	82%	513	90%	0.08
**Health-related**						
Use of specialist care in the last 2 years	Yes	74	85%	480	84%	0.81
Use of psychiatric care in the last 2 years	Yes	38	44%	286	50%	0.27
Use of medication	Yes	71	83%	473	83%	0.95
Depressive disorder[6]	0-21	9.29	4.60	7.65	4.52	0.002
Anxiety disorder[6]	0-21	8.95	4.31	7.96	4.15	0.04
Severity of complaints[7]	5-30	11.56	4.73	11.07	4.52	0.34
Physical health[8]	0-100	33.87	8.91	34.04	9.46	0.88
Mental health[8]	0-100	35.89	13.01	38.98	12.94	0.04
Health compared to a year ago[8]	Worse	34	39%	207	36%	0.62
Hypochondria[9]	0-14	6.29	3.41	5.32	2.99	0.01
Symptom scale[5]	20-80	42.79	11.60	41.21	10.24	0.19
Coping strategies[5]	17-68	40.34	10.16	41.13	9.37	0.47
Subjective health complaints (SHC)	Yes	17	22%	86	17%	0.21
**Self-perceived ability**						
Positive expectations for staying at work	Yes / Inconclusive	46	53%	351	62%	0.13
Disability[5]	7-28	21.13	4.97	20.56	4.98	0.33

Work ability in general[10]	0-10	4.34	2.24	4.60	1.99	0.28
Work ability in the context of work load[10]	2-10	5.86	1.71	6.11	1.51	0.16
Possibilities for returning to work[11]	0-36	15.04	8.58	15.30	9.59	0.81

Footnotes:

[1]No fully documented work status during follow-up

[2]No = number

[3]Fully documented work status during follow-up

[4]SD = standard deviation

[5]Based on the work and well-being inventory questionnaire (WBI)

[6]Based on the hospital anxiety and depression scale (HADS)

[7]Based on the patient health questionnaire (PHQ-15)

[8]Based on the short form health survey 36 (SF-36)

[9]Based on the Whitely index questionnaire (WI)

[10]Based on the work ability index (WAI)

[11]Based on the obstacles to return to work questionnaire (ORQ)

Table S4. Multivariable logistic regression multiple imputation analysis (pooled data) of all final potential predictors for staying at work for participants with subjective health complaints (SHC) and other disorders separately.

		**SHC (No[1]=86)**			**Other disorders (No=433)**		
**Domains**	**Categories/Ranges**	**OR[2]**	**95% CI[3]**	*p*	**OR**	**95% CI**	*p*
Socio-economic and work-related							
Previous absenteeism for the same health complaint	No	Reference			Reference		
	Yes	0.29	0.10-0.85	0.02	0.69	0.44-1.09	0.11
Work disability benefits	No / Partial	Reference			Reference		
	Full	0.06	0.01-0.57	0.01	0.13	0.08-0.22	0.000
Health-related							
Mental health[4]	0-100	1.07	1.02-1.13	0.01	1.03	1.01-1.05	0.001
Coping strategies[5]	17-68	1.10	1.02-1.19	0.01	1.02	1.00-1.04	0.15
Self-perceived ability							
Positive expectations for staying at work	No	Reference			Reference		
	Yes / Inconclusive	4.44	1.47-13.45	0.01	2.88	1.85-4.49	0.000

Footnotes:

[1]No = number

[2]OR = odds ratio

[3]95% CI = 95% confidence intervals

[4]Based on the short form health survey 36 (SF-36)

[5]Based on the work and well-being inventory questionnaire (WBI)
